# Analysis and control of the photon beam position at PLS-II

**DOI:** 10.1107/S1600577516001338

**Published:** 2016-02-18

**Authors:** J. Ko, I.-Y. Kim, C. Kim, D.-T. Kim, J.-Y. Huang, S. Shin

**Affiliations:** a Pohang Accelerator Laboratory, POSTECH, Pohang, Kyungbuk 790-784, South Korea

**Keywords:** photon beam position monitor, stability, SVD analysis

## Abstract

At third-generation light sources, the photon beam position stability is a critical issue for user experiments. In general, photon beam position monitors are developed to detect the real photon beam position, and the position is controlled by a feedback system in order to maintain the reference photon beam position. At Pohang Light Source II, a photon beam position stability of less than 1 µm r.m.s. was achieved for a user service period in the beamline, where the photon beam position monitor is installed. Nevertheless, a detailed analysis of the photon beam position data was necessary in order to ensure the performance of the photon beam position monitor, since it can suffer from various unknown types of noise, such as background contamination due to upstream or downstream dipole radiation, and undulator gap dependence. This paper reports the results of a start-to-end study of the photon beam position stability and a singular value decomposition analysis to confirm the reliability of the photon beam position data.

## Abstract 2

The variation of the photon beam position in a beamline, which is a critical issue for user experiments, is analyzed and corrected through the correlation link with the electron beam position in the storage ring.

## 1. Introduction   

After the completion of the Pohang Light Source II (PLS-II) project to upgrade the Pohang Light Source (PLS) on 21 March 2012, PLS-II (Shin *et al.*, 2013 ▸) is now in full operation. As a result of the upgrade, the PLS beam energy increased from 2.5 GeV to 3.0 GeV, and the stored beam current increased from 200 mA to 400 mA. The emittance is improved from 18.9 nm at 2.5 GeV to 5.8 nm at 3 GeV, while the PLS storage-ring tunnel structure remains unchanged. In addition, top-up mode operation is used to stabilize the stored electron beam orbit and the synchrotron radiation flux. Currently, a total of 31 beamlines including 18 insertion device beamlines are in operation for user service.

One of the major beam operation issues in the storage rings of third-generation light sources is the beam position stability for the photon beam as well as the electron beam. Therefore, PLS-II accommodated 96 newly designed beam position monitor (BPM) pickups and new digital BPM electronics (Libera Brilliance^1^) and achieved an electron beam stability of 1 µm r.m.s. The ultimate goal of the beam stability in third-generation light sources is to deliver stable photon beam to the beamline users. Unfortunately the photon beam stability can be degraded while passing through the beamline in spite of the stable electron beam from the BPM installed in the storage ring. This invokes the need for a photon beam position monitor (PBPM) as a reference in the beamline.

The PBPM has been widely used for photon beam position measurements. It provides photon beam position information with stable micrometer resolution. In addition to its powerful ability, the most common PBPM has a simple structure equipped with blades (symmetric in the transverse direction) to cut a small part of the photon beam. Then, the photo-current can be measured from the blades, using the photoelectric effect. The current difference between the upper and the lower blades provides information such as the electron beam position. Unfortunately, when it is used in an undulator beamline, a PBPM can suffer from background contamination due to dipole radiation. Therefore a thorough demonstration of the reliability of this PBPM is required.

In this paper we analyze the PBPM measurements to investigate the correlation among electron BPM (e-BPM), PBPM and beamline flux and to find the cause of long-term photon beam position drift by using a singular value decomposition (SVD) analysis with quantitative approach. §2 introduces the PBPM system of PLS-II. §3 describes an investigation of the correlation among e-BPM, PBPM and beamline flux and the result of the SVD analysis. Control of the photon beam trajectory is described in §4, and §5 presents our conclusions.

## 2. PLS-II PBPM   

**Figure 1:**
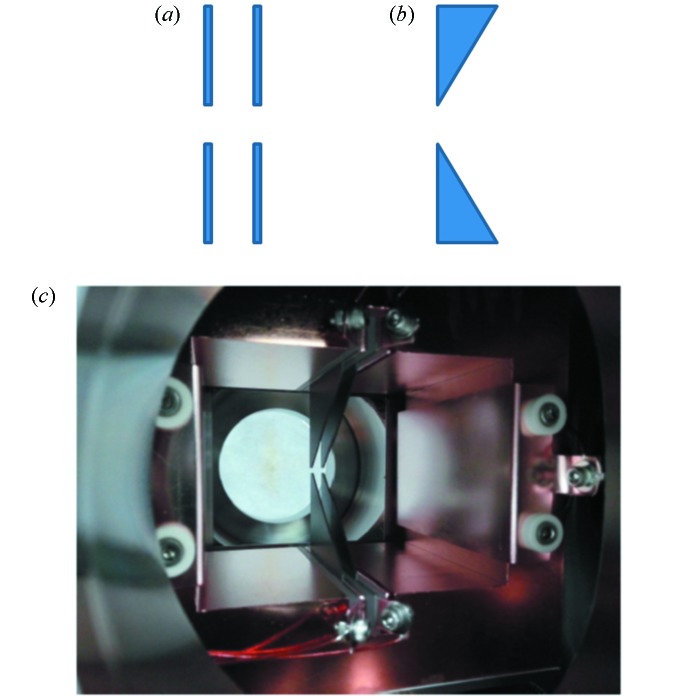
Four-blade type PLS-II PBPM pick-up system. (*a*) Front view of the blades. (*b*) Side view of the blades. (*c*) Photograph of the four-blade pick-up system.

The PLS-II PBPM system (Kim *et al.*, 2010 ▸) consists of pick-up, translation device and Libera photon electronics. Fig. 1 ▸ shows the pick-up for the PLS-II PBPM. There are two types of pick-ups: two-blade type and four-blade type. For the blades, 0.5 mm-thick tungsten plates are used, which are installed on the top and bottom of the detector head. The detector head is a rectangular pipe made of copper, through which the radiation passes. Sapphire plates are inserted between the blades and the detector head for electrical insulation and good thermal conduction simultaneously. On both sides of the detector head are installed high-voltage electrodes to remove stray particles inside the detector head. The detector head is connected to a water-cooling system to keep the temperature constant in each part of the detector head.

**Figure 2:**
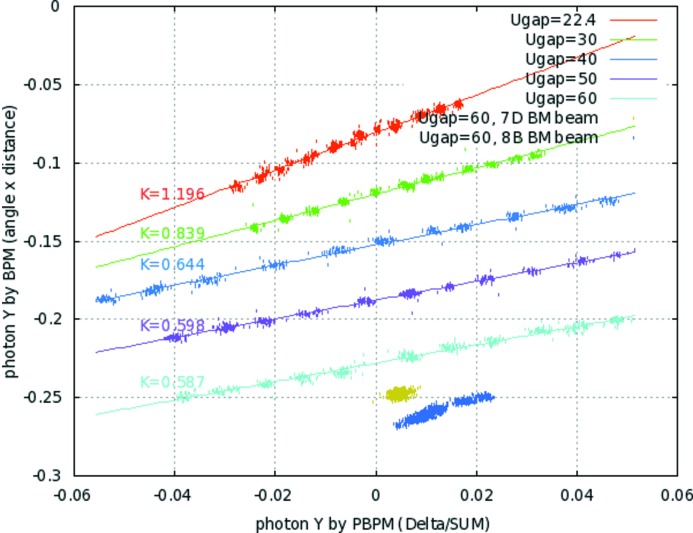
Variation of the calibration factor along the undulator gap. The bending radiation effect is also measured. The horizontal axis indicates the PBPM reading and the vertical axis indicates the electron beam steering.

**Figure 3:**
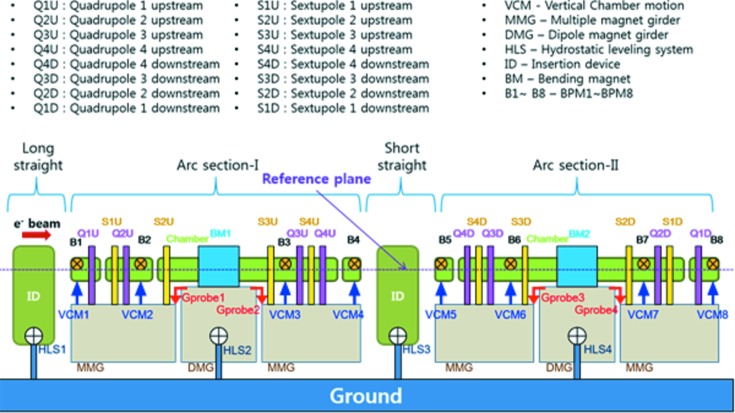
Geometric structures around the undulator for one cell. The PLS-II storage ring consists of a total of 12 cells.

The PBPMs are installed in the front-end of each beamline. Horizontal and vertical translation devices were installed on a stable stand. The PBPM chamber can be moved in the transverse direction of the radiation by using these devices. The first calibration was performed using synchrotron radiation. By moving the translation device, the photo-currents measured from the upper and the lower blades are used in the formula 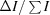, which gives information on the position change of the beam. Fig. 2 ▸ shows the results of the second calibration using electron beam steering. The second calibration determines the ratio between the photon beam position measured in the PBPM and the photon beam position calculated by steered electron beam orbit. The calibration factors were measured at different undulator gaps in order to investigate the beam profile effect of the undulator gap and bending radiation contamination. The electron beam was steered with local bumps at the radiation source points for both undulator and bending magnets. Geometric structures around the undulator including upstream bending magnet, correctors and BPMs are shown in Fig. 3 ▸. The effect of the upstream bending radiation is negligible on the PBPM measurement in the undulator beamline, showing good linearity along the electron beam steering for each undulator gap in Fig. 2 ▸. The calibration factor decrease along the undulator gap is caused by radiation beam profile changes. However, a gap feedforward table is used to keep the same calibration factor along each undulator gap.

**Figure 4:**
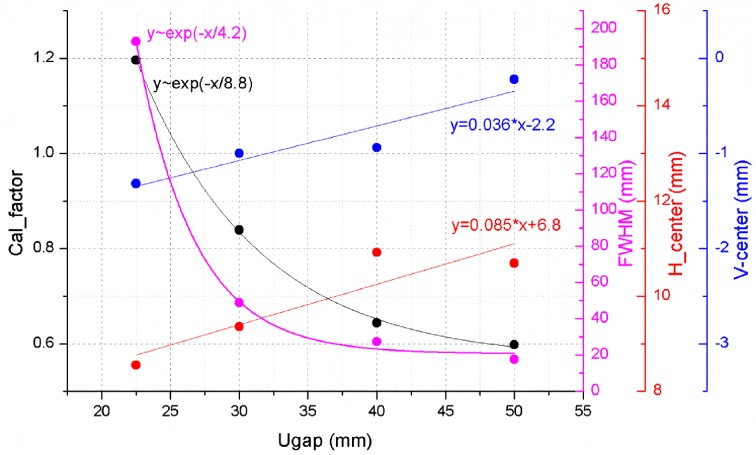
Horizontal photon beam size, calibration factor and photon beam position as a function of undulator gap. Both calibration factor and photon beam size are proportional to the exponential function.

To investigate the calibration factor decrease effect with undulator gap, the radiation beam profiles are scanned along the undulator gap. Generally, the radiation beam size decreases with undulator gap due to a transverse deflection decrease of the electron beam along the undulator gap. The expression 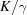 is defined as the maximum slope of the transverse deflection caused by the undulator. Increasing the undulator gap is the source of the *K* decrease. Here *K* is the deflection parameter and is given by
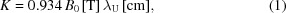
where 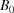 is the magnetic field, which is proportional to 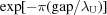. Notice that the vertical photon beam size remains relatively constant due to no deflection change along the undulator gap (Schlax, 2010 ▸). Fig. 4 ▸ shows the horizontal photon beam size, calibration factor and photon beam position along the undulator gap. Both the calibration factor and the photon beam size are proportional to the exponential function. The result shows that the calibration factor decrease with the undulator gap is caused mainly by a change in the effective photon beam size.

## 3. Correlations among e-BPM, PBPM and beamline flux   

After installing and calibrating the PBPM system, a verification of the performance of the PBPM is required in order to use reliable photon beam position data. We analyzed the PBPM data in two main areas. First, we investigated the short-term correlation among e-BPM, PBPM and beamline flux. Secondly, we examined the cause of the long-term drift of the photon beam position. To explore the position drift source we used SVD analysis with a quantitative approach. As a major part of model-independent analysis, a spatial-temporal mode analysis technique was applied in order to identify the source of the position drift.

**Figure 5:**
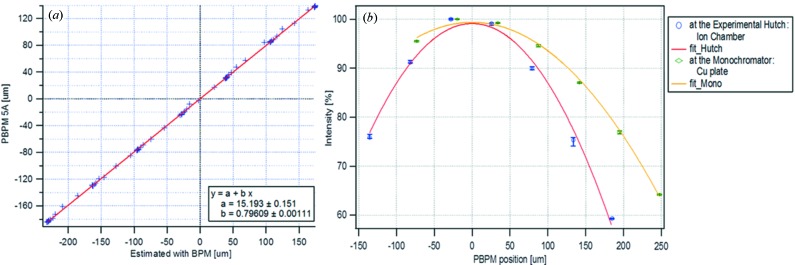
(*a*) Correlation between e-BPM and PBPM. (*b*) Correlation between PBPM and flux in the beamline.

The correlation was measured by steering the electron beam orbit at the source point. At the same time, the photon beam position from the PBPM and flux at the beamline were also measured. In Fig. 5(*a*) ▸, the horizontal axis indicates the estimated photon beam position from upstream and downstream e-BPMs of the undulator. A strong linear correlation is shown between the position estimated from e-BPMs and the position measured from the PBPM, but a 20% calibration error was found due to a PBPM motor calibration error. Fluxes at the monochromator and the experiment hutch in the beamline were measured for each photon beam position. These results show good evidence of a reliable performance of the PBPM on a short-term time scale.

**Figure 6:**
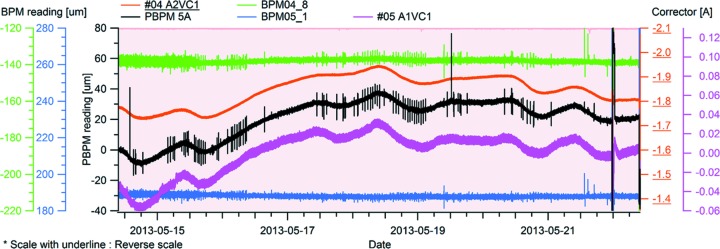
PBPM (black), e-BPM (lime and blue), corrector (upstream: orange; downstream: purple) and beam current (background pink) variations during user operation (eight days).

During long-term time scale user operation, the strong correlation was broken. Ground deformation was found later to be the cause. Fig. 6 ▸ shows the variation of the photon beam position during user operation. Despite feedback freezing of the electron beam position at the BPMs, the photon beam position at the PBPM varied by up to 30 µm. However, as shown in Fig. 6 ▸, the photon beam position had a strong correlation with the orbit correctors that are included in the slow orbit feedback system and installed in the upstream and downstream undulator. This strong correlation between PBPM and the corrector data verifies that the correctors installed in the slow orbit feedback system are functioning correctly to compensate for BPM displacement that occurs in real time during user operation. Here, the beam current dependency of the BPMs was ignored due to top-up operation, and it was found that the BPM displacement is caused by ground deformation.

In order to demonstrate the ground deformation effect on corrector variation during user operation, SVD analysis was applied. In general, SVD of the data matrix containing the beam position yields a spatial-temporal mode analysis of beam motion by effectively accomplishing statistical principal component analysis. Mathematically, the SVD of a matrix *B* yields (Wang, 2003 ▸)
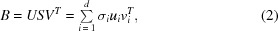
where 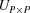 = 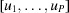 and 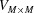 = 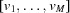 are orthogonal matrices, 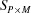 is a diagonal matrix with non-negative 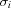 along the diagonal in decreasing order, *d* = 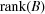 is the number of non-zero singular values, and the vectors 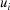 and 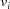 are the *i*th left and right singular vectors, respectively. Each set of {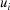, 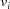} defines a spatial-temporal mode, where 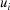 gives the temporal variation and 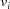 gives the spatial variation. The singular values reveal the system dimensionality and relative magnitudes, while each set of singular vectors forms an orthogonal basis of the various spaces of the matrix.

**Figure 7:**
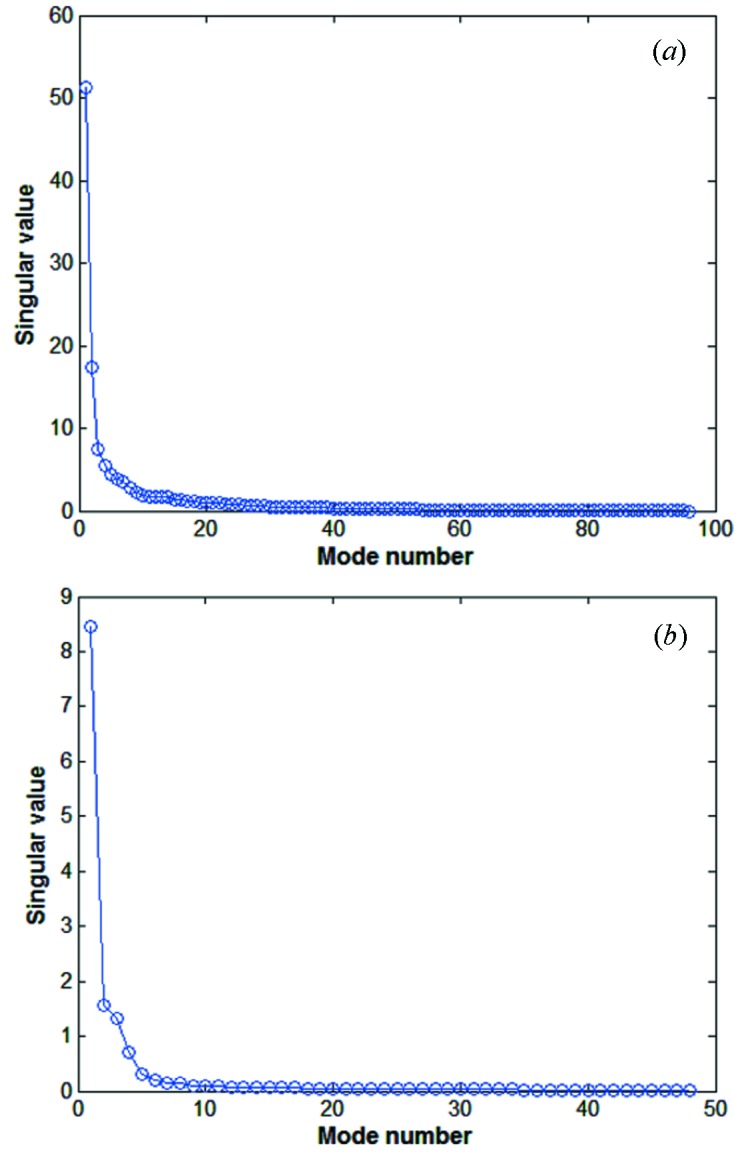
Singular value plots of the SVD results from (*a*) correctors and (*b*) HLSs.

**Figure 8:**
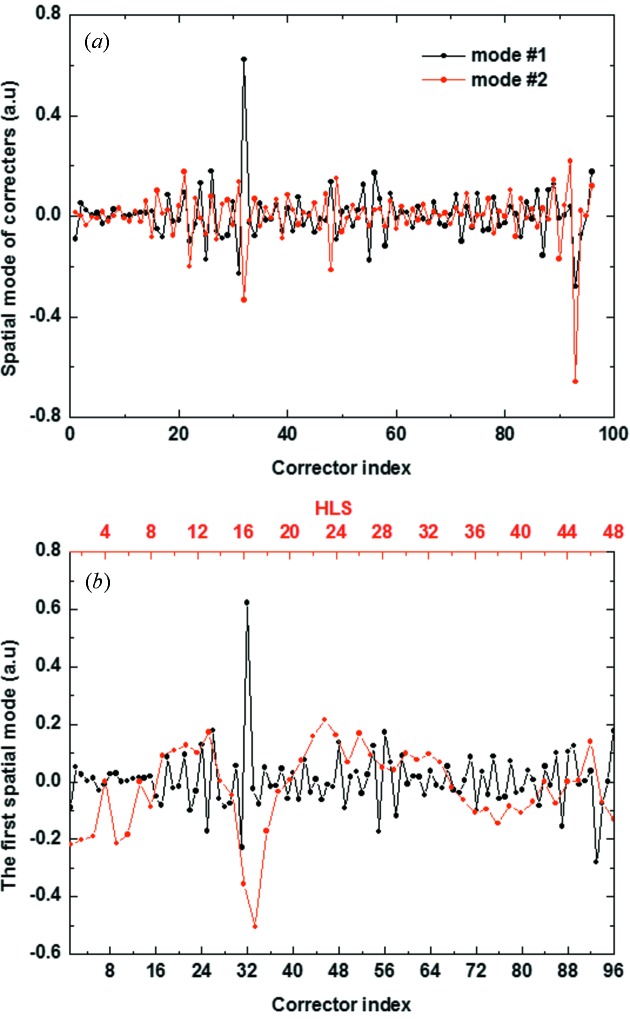
Spatial eigenvector plots of the SVD result. (*a*) The first two dominant spatial eigenvectors from corrector readings. (*b*) The first spatial eigenvector for HLS readings and corrector readings.

We performed SVD analysis for two data matrices containing the corrector set values and ground deformation data from the hydrostatic leveling system (HLS) (Seryi *et al.*, 2001 ▸). Two matrices of 135000 samples each for the 96 correctors and 48 HLSs are taken in the SVD analysis. Here 135000 samples correspond to a 37.5 h time scale. The diagonal element of the singular matrix *S* provides an estimate of the modes. Fig. 7 ▸ shows that a few modes of these singular values are considerably larger than others. In particular, the first singular value of each matrix is predominantly large. This indicates that there is major motion of each matrix. Fig. 8(*a*) ▸ shows the first two spatial eigenvectors from matrix *B* of corrector readings. In the general case of BPM readings, the first and second eigenvectors correspond to ‘sine-like’, ‘cosine-like’ or ‘dispersion-like behaviors since the general beam motion in the storage ring consists of betatron oscillation and energy-dependent orbit. But, unlike the general case of BPM readings, slow orbit drift by perturbation source affects the spatial mode pattern from matrix *B* of corrector readings in the feedback system. There is a large perturbation source around corrector index 30 for the first dominant eigenvector in Fig. 8(*a*) ▸. The main perturbation source around corrector index 30 is ground deformation, deduced by the first spatial eigenvectors from matrix *B* of HLS readings in Fig. 8(*b*) ▸.

**Figure 9:**
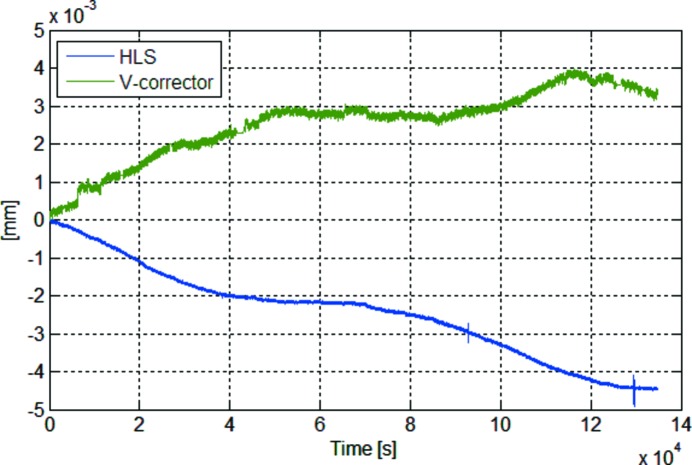
Temporal waveforms for the first modes of HLSs and correctors.

Temporal mode waveforms for the first value are shown in Fig. 9 ▸. It should be noted that there is a strong correlation between the two temporal waveforms. To quantify the correlation, we used the correlation coefficient and calculated it to be about −0.94. Here the correlation coefficient is given by
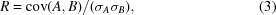
where cov is the covariance and 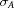 is the standard deviation of *A*. This strong correlation means that the corrector set value in the feedback system is changed to correct for the slow orbit drift caused by ground deformation as the perturbation source.

## 4. Control of the photon beam trajectory   

The long-term photon beam position in the beamline can drift due to environmental changes in spite of the precise control of the electron orbit. Systematic effects, such as a small temperature dependence of the electron BPM electronics in the technical gallery and movements of the e-BPM blocks in the storage ring caused by ground deformation, may lead to a change of the photon beam position in the beamline at the few tens of micrometers level. To realise a stable photon beam trajectory, a slow photon beam position feedback system, which consists of slow electron orbit feedback system and local electron orbit solver program, was implemented at PLS-II. Because only one PBPM is available at the PLS-II beamline, the photon beam position change is compensated by a pure angle variation of the orbit at the source point. The electron orbit change in two e-BPMs, 1 and 2, at both ends of the source point to restore a deviated position from the target value in the PBPM is given by
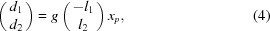
where *g* is the general gain factor including the geometry factor, 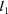 and 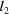 are e-BPM locations from the source point, and 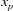 is a deviated position from the target value in the PBPM. Note that the electron orbit from the quadrupole center is defined by
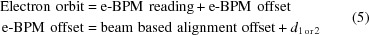
The local electron orbit solver program updates the e-BPM offset every 2 s by solving for 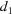 and 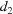 in equation (4). Here, the beam based alignment offset is fixed during the user run. Then, the slow electron orbit feedback system corrects the photon beam position to the target value at the PBPM by changing the local electron orbit in equation (5). The main advantage and characteristic of this scheme for photon beam position feedback is that it does not need to modify the existing slow electron orbit feedback system and does update the e-BPM offset rather than the electron reference orbit. The PBPM feedback will only be active if the gaps are closed and are below the predefined thresholds of the beam current, beamline shutter, electron beam r.m.s. values and PBPM deviation.

**Figure 10:**
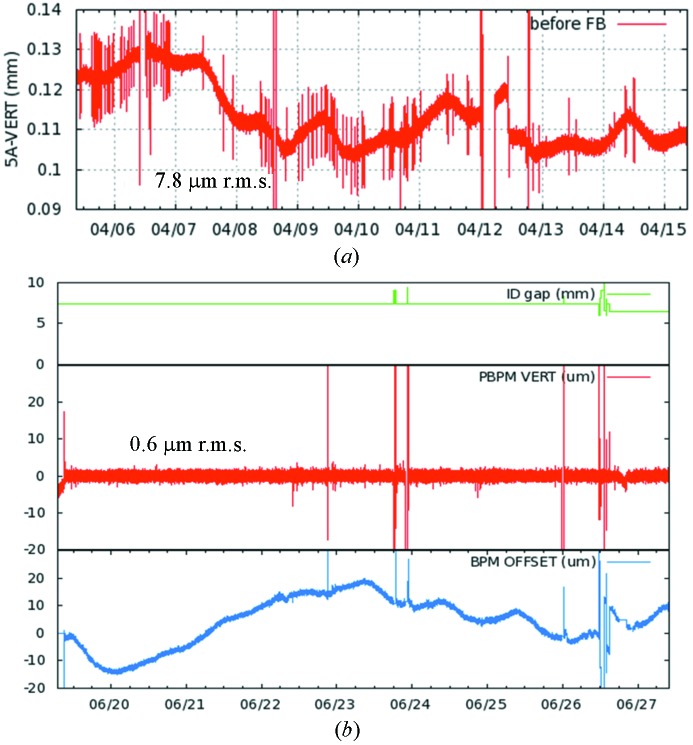
(*a*) Photon beam trajectory variation without the PBPM feedback system during user run (ten days). (*b*) Stable photon beam trajectory by the slow PBPM feedback.

Fig. 10 ▸ depicts the variation of the upstream e-BPM together with the corresponding stabilized PBPM readings during top-up operation. The photon beam variation without PBPM feedback is also compared in the figure. The resulting temporal distributions of the photon beam positions exhibit r.m.s. values of 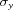 = 0.6 µm for eight days. The temporal distributions of the BPM offset to correct the photon beam position to the target show a long-term drift trend combined with a day-by-day variation of ∼30 µm.

## 5. Conclusion   

We analyzed and controlled the photon beam position at PLS-II and confirmed a strong short-term correlation of the PBPM with the e-BPM and flux at the beamline. However, during long time scale user operation the strong correlation breaks due to e-BPM displacement by ground deformation. The strong correlation observed between corrector values and PBPM values implies that the correctors in the slow orbit feedback system are working to compensate for physical BPM displacement in user operation. A SVD analysis of the temporal drifts of the dominant modes revealed that ground deformation causes the changes in the corrector set value by the BPM displacement. However, this photon beam position drift during user operation was corrected and kept in the 1 µm r.m.s. range by using the photon beam position feedback system.

## Footnotes

1 See http://www.i-tech.si/accelerators-instrumentation/libera-brilliance-plus/benefits_1 for the high-resolution Libera BPM.
